# Impact of personality traits on start-up preparation of Hong Kong youths

**DOI:** 10.3389/fpsyg.2022.994814

**Published:** 2022-10-28

**Authors:** Jiahao Zhuang, Rui Xiong, Hongyi Sun

**Affiliations:** Department of Advanced Design and Systems Engineering (ADSE), City University of Hong Kong, Kowloon, Hong Kong SAR, China

**Keywords:** entrepreneurial intention, human capital theory, start-up preparation, social networking, personality traits, theory of planned behavior

## Abstract

Entrepreneurship is a tool for driving economic and social progress. Especially in Hong Kong, the government has recently taken steps to encourage young people to engage in entrepreneurship. However, Hong Kong youths’ entrepreneurial intentions are still low. The objective of this study is to empirically explore the impacts of personality traits on start-up preparation among Hong Kong youths through the constructs of the theory of planned behavior (TPB). Through a multi-channel survey, we finally collected 230 valid respondents aged 18 to 40. In addition, this study used SmartPLS software to conduct confirmatory factor analysis for the measurement model as well as path analysis for the structural model. This study’s results suggested that creativity, risk-taking propensity, need for achievement, and internal locus of control influence TPB models’ components and indirectly influence start-up preparation through TPB models’ components. Also, attitude and perceived behavioral control influence intention, and intention influences preparation. Furthermore, prior entrepreneurial experience and entrepreneurship education positively influence preparation. In conclusion, this study revealed the mediating effects of TPB components between four personality traits and start-up preparation. Finally, this study had theoretical implications by providing the influence of six personality traits on youths’ entrepreneurial intention and preparation through the TPB model and the human capital theory. This study also had practical implications by providing suggestions for the government and higher education institutions.

## Introduction

Entrepreneurship has emerged as a critical driver of economic and social progress around the world (Audretsch, 2004), such as job creation. Policymakers are starting to recognize the significance of entrepreneurship. As a result, governments worldwide regard entrepreneurship as the most important component of economic success (Hébert and Link, 2009). In this vein, there has been increased interest in entrepreneurship from academic institutions, government policymakers, and business sectors in Hong Kong (Lerner, 2010). Specifically, the Hong Kong Special Administrative Region (HKSAR) launched *Funding Scheme for Youth Entrepreneurship in the GBA* by providing incubation services and funding to young ventures in Hong Kong, Macao, and nine cities in Greater Bay Area. Moreover, Non-governmental organizations, businesses, and the government launched *Space Sharing Scheme for Youth* in 2017 to provide a low-cost office for young entrepreneurs. Despite the efforts by the HKSAR, the Hong Kong start-up ecosystem only ranked 31st, far behind Beijing (4th). Thus, it is urgent for us to examine entrepreneurship in Hong Kong. There are various factors that influence entrepreneurship. In this study, we explored Hong Kong entrepreneurship from a trait approach.

Although personality traits have been extensively studied in the field of entrepreneurship, we found that there are some gaps in the field (Taormina and Lao, 2007; Nabi and Liñán, 2013). First, although there are lots of personality traits, they are examined individually (Ng et al., 2019). Second, most previous research focuses on intention only (Rosique-Blasco et al., 2018). There is an urgent need to move downstream to investigate preparation. Third, previous studies mainly focused on the sample of students (Eid et al., 2019; Maheshwari, 2021). Finally, the effects of variables of human capital theory on start-up preparation remain unclear. To narrow these gaps, this study aims to empirically explore the impacts of six personality traits on start-up preparation (SP) among Hong Kong youths through the constructs of the theory of planned behavior (TPB).

The novelty of this paper covers four folds. First, this study explored six personality traits at the same time rather than individually. Second, this study moved downstream to explore the relationship between entrepreneurial intention and start-up preparation. Furthermore, this study explored the mediating role of TPB models’ constructs between personality traits and start-up preparation. Third, the subjects of this study are young people from 18 to 40 years old rather than university students. Fourth, following the suggestion by Marvel et al. (2016), this study adopted human capital theory by Schultz (1961) and Becker (2009) to build the conceptual model.

Our study contributes to personality traits literature in the field of entrepreneurship. First, prior personality traits research mainly focused on the direct influences of personality traits on the entrepreneurial intention stage. Also, a few studies have started to pay attention to the indirect effects by examining the personality traits, but individually. We contribute to this literature stream by empirically moving downstream to study the start-up preparation stage and test the mediating role of TPB models’ constructs with a focus on six personality traits. Second, the literature on personality traits mainly focuses on university students (Eid et al., 2019; Maheshwari, 2021), while this study adds to the existing knowledge by covering a wider age group, namely, young people aged 18 to 40. Third, previous scholars have studied the students’ entrepreneurial intention and most studies only considered the demographic variables as control ones (Maheshwari, 2021). The current paper tries to advance empirical research on youths’ entrepreneurship by adopting the human capital theory. In this vein, our analysis contributes to a better understanding of the role of entrepreneurship education, previous entrepreneurial experience, and work experience in the entrepreneurial process of young people.

Theoretical and practical implications will be explored finally.

## Literature review and hypothesis development

Entrepreneurship is an interdisciplinary topic (Anderson et al., 2007). In this study, we explored personality traits. In this session, we undertook a literature review on the study of personality traits in entrepreneurship research.

### Research of personality traits

The personality traits of research in the entrepreneurship field have been widely studied. There is a large body of personality traits. For instance, Taormina and Lao (2007) examined three (achievement striving, social networking, and optimism) personality traits in three groups of people in Guangzhou, namely people without entrepreneurial intention, people with entrepreneurial intention, and successful entrepreneurs. They revealed that personality traits strongly influence potential entrepreneurs and the environment strongly influences successful entrepreneurs. From a cross-national study, Prabhu et al. (2012) found self-efficacy moderates the relationships between proactive personality and (high growth and lifestyle) two kinds of entrepreneurial intentions. Bolton and Lane (2012) developed a measure of individual entrepreneurial orientation from three personality traits. Based on the sample of two European countries, Nabi and Liñán (2013) found that there is an indirect influence of risk perception on entrepreneurial intention. In India, Roy et al. (2017) found that perceived self-efficacy mediates and moderates personality traits and entrepreneurial intention. Similarly, data on 1,126 Spanish university students by Rosique-Blasco et al. (2018) revealed the mediating role of attitude and entrepreneurial self-efficacy (ESE) between four personality traits (creativity, proactivity, risk aversion, and internal locus of control) and intention. Munir et al. (2019) found the distinct mediating effects of three personality traits (risk-taking propensity, proactivity, and internal locus of control) and EI in China and Pakistan. From multiple perspectives, Ng et al. (2019) examined the indirect effects of proactivity, entrepreneurship education, and opportunities on EI through TPB models’ components. By combing the TPB model and entrepreneurial event model (EEM), Eid et al. (2019) revealed the effects of two personality traits (autonomy and creativity) on perceived behavioral control, perceived desirability, and subjective norms. A recent study by Maheshwari (2021) compared the direct effects of educational support, individual personality traits, and TPB models’ components on EI, which finally revealed that the TPB models’ components are the most influential factors in EI.

Based on the review, research on personality traits covers quite different variables (See Table 1), including proactivity (PROA) (Prabhu et al., 2012; Munir et al., 2019), locus of control, need for achievement (NA), risk-taking propensity (RTP) (Nabi and Liñán, 2013), tolerance of ambiguity (TA) (Rosique-Blasco et al., 2018), self-confidence (SC) (self-efficacy) (Maheshwari, 2021), creativity (CREA), social networking (SNET) (Taormina and Lao, 2007), optimism (OPT), and autonomy (AUTO) (Eid et al., 2019).

**Table 1:** Research on personality traits and entrepreneurial intention.

Ref.	Region	Sample	TPB model	Personality traits	EI
Taormina and Lao (2007)	Guangzhou, China	Three group respondents		NA SNET OPT	√
Prabhu et al. (2012)	China, Finland, Russia, and the USA	233 college students	√	PROA	√
Bolton and Lane (2012)	USA	1,102 students	√	PROA RTP CREA	√
Nabi and Liñán (2013)	Spain and Great Britain	619 European	√	RTP	√
Roy et al. (2017)	India	476 university graduates	√	LOC RTP CREA	√
Rosique-Blasco et al. (2018)	Spain	1,126 university students	√	LOC NA RTP TA	√
Munir et al. (2019)	China and Pakistan	1,016 students	√	PROA NA RTP	√
Ng et al. (2019)	Malaysia	209 university students	√	PROA	√
Eid et al. (2019)	UAE's seven emirates	688 senior university students	√	CREA AUTO	√
Maheshwari (2021)	Vietnam	164 university students	√	NA TA SC	√

By reviewing previous studies, as shown in Table 1, this study found some research gaps to narrow:

1. Although some recent studies have focused on the mediating role of TPB’s antecedents and personality traits, personality traits were examined individually in these studies (Roy et al., 2017; Ng et al., 2019).
2. Fayolle and Liñán (2014) called for exploring the intention-behavior relationship. However, most studies only focused on the entrepreneurial intention stage.
3. Intention models need to be tested on a diverse range of people at various phases of their lives (Peterman and Kennedy, 2003). However, most studies only focused on the sample of students (Eid et al., 2019; Maheshwari, 2021).
4. Marvel et al. (2016) suggested that future studies investigate the many dimensions of human capital at various phases of the entrepreneurial process. As past research is limited to student samples, the influences of work experience and entrepreneurial experience on start-up preparation are unclear.

Therefore, to narrow these gaps, the novelty of this paper is four-fold. First, regarding the TPB model, we explored six personality traits (creativity, need for achievement, locus of control, risk-taking propensity, tolerance of ambiguity, and social networking at the same time). Moreover, this study revealed the mediating effects of the constructs of the TPB on the relationship between personality traits and start-up preparation. Second, this study moved downstream to explore the relationship between entrepreneurial intention and start-up preparation. Third, we surveyed youths aged 18 to 40 instead of the student sample. Fourth, based on the youth group, we adopted the three most cited variables suggested by Marvel et al. (2016) to test the human capital’s effects on the youth. Work experience, entrepreneurship education, and prior entrepreneurial experience are the three most common dimensions of human capital. The principal contribution of this research is to examine the impact of six personality traits (CREA, NA, RTP, TA, LOC, SNET) on entrepreneurial intention (EI) and start-up preparation (SP) of youth group aged 18 to 40 based on the TPB model by Ajzen (1991) and human capital theory.

### The definitions of six personality traits

There are explanations of six personality traits. Specifically, “creativity” (CREA) refers to the capacity to generate new and useful ideas (Amabile et al., 1996). Need for achievement (NA) refers to one’s responsibility for participating in actions to attain one’s desired goal (McClelland, 1961). Locus of control (LOC) refers to a person’s generalized expectations about their capacity to govern their life (Begley and Boyd, 1987). Risk-taking propensity (RTP) is defined as taking uncertain decisions or actions, irrespective of the outcomes (Jackson, 1977). Ambiguity tolerance (TA) is described as a person’s perception of and response to an unclear situation (Stanley Budner, 1962). Social networking (SNET) refers to the proclivity to form relationships and communicate with others and it is one of the most basic human needs (Maslow, 1981).

### The theory of planned behavior and human capital theory

The theory of planned behavior (TPB; Ajzen, 1991), an influential social psychology theory, is used to examine the relationship between an individual’s beliefs and actions. Ajzen, a notable scholar in the field of social psychology, adapted an existing theory called the Theory of Reasoned Action (TRA; Fishbein and Ajzen, 1975), and introduced the concept of planned behavior in 1985. He proposed that an individual’s desire to engage in a given action can predict their decision to engage in that behavior (Ajzen, 1985). According to Ajzen (1991), there are three antecedent variables. First, entrepreneurial attitude (EA) refers to the degree of individual evaluation of starting a business. Second, subjective norms (SN) refer to the extent of individuals’ perception of social approval from significant others, such as family members, friends, peers, or close relatives, of starting a business. Third, perceived behavioral control (PBC) refers to the individuals’ perceived ease or difficulty in starting a business. This study moved downstream to explore both entrepreneurial intention and preparation. Specifically, entrepreneurial intention (EI) refers to a strong desire to start a new company (Krueger and Brazeal, 1994). Start-up preparation refers to the entrepreneurial preparations that include conducting market research, searching for funding, making a business plan, and gathering information about the procedures of starting a business (Van Auken and Neeley, 2000).

We also adopted the human capital theory, which is critical to entrepreneurship (Shane, 2000; Ardichvili et al., 2003). The human capital theory brought out by Schultz (1961) and Becker (2009) was initially used to study the value of education and emphasizes the importance of a person’s education, experience, and knowledge. Human capital facilitates entrepreneurs to take advantage of opportunities by gaining financial resources and creating new businesses (Bruns et al., 2008), and even achieving entrepreneurial success (Unger et al., 2011).

### Entrepreneurial intention and three antecedents of TPB model

The TPB model has been widely adopted to study intention and behavior in many domains, such as psychology, social sciences, and management. In the field of entrepreneurship, a wide range of research has adopted this theory to study entrepreneurial behavior (Marques et al., 2012; Fayolle and Liñán, 2014). Earlier research has confirmed this relationship empirically, showing that a person’s entrepreneurial attitude positively influences their entrepreneurial intentions (Krueger et al., 2000). Also, previous research has revealed that SN has a direct effect on EI (Gird and Bagraim, 2008; Schlaegel and Koenig, 2014). However, Krueger et al. (2000) indicated that the influence of social support on EI was not identified in American students. Also, Autio et al. (2001) stated that there was no indication that social support influenced EI in American students. In addition, the relationships among the TPB model have been widely examined in previous empirical studies. For example, Carr and Sequeira (2007) found past family business exposure influence EI through EA, SN, and PBC. Based on the discussion, we proposed the hypotheses:

*H1:* (a) EA and (b) PBC, (c) SN positively and significantly influence EI among Hong Kong youths.

### Entrepreneurial intention and start-Up preparation

Individual’s commitment to doing a certain activity that makes entrepreneurship viable (LeBrasseur et al., 2003). Regarding the TPB model, the component of intention is represented by “entrepreneurial intention,” and that of behavior is meant by “start-up preparation” (Ajzen, 1991). Previous empirical studies have confirmed entrepreneurial intention is an important predictor of start-up action (Bird, 1988; Krueger et al., 2000). For example, Kautonen et al. (2015) revealed an association between EI and future start-up behavior. Similarly, Shinnar et al. (2018) found a high correlation between business venture intention and actual behavior. However, studies combining personality traits and examining the relationship between EI and entrepreneurial behavior remain scarce. This study uses start-up preparation (SP) to measure entrepreneurial behavior. Based on the discussion, we proposed the hypotheses:

*H1d:* EI positively and significantly influences on SP among Hong Kong youths.

### Creativity

Entrepreneurs are usually seen as creative individuals, and creativity is viewed as an important aspect of entrepreneurship (Zhao et al., 2010). Previous research has demonstrated that creativity promotes intention (Altinay et al., 2012; Chaudhary 2017) and entrepreneurship success (Hamidi et al., 2008). Accordingly, Feldman and Bolino (2000) argue that those who are seen to be more creative are more likely to start their own business. Using the TPB model, researchers discovered that creativity has a favorable influence on entrepreneurial attitudes (Zampetakis, 2008; Laguía et al., 2019). Furthermore, more creative youths will have more business ideas, be able to analyze market wants, and conceptualize their entrepreneurial initiatives, whereas youths will be unable to start and lack confidence in entrepreneurial operations if they lack innovation and ideas. Based on the discussion, we proposed the hypotheses:

*H2:* CREA positively and significantly influences (a) EA and (b) PBC among Hong Kong youths.

### Need for achievement

McClelland (1961) need for achievement (NA) theory is one of the most well-known theories in entrepreneurship studies. McClelland (1961) discovered the link between a person’s desire to succeed and their desire to establish a business. Since then, most research has revealed the positive and significant effects of the NA on the intention to start a venture (Gürol and Atsan, 2006). In this case, we believe those with a strong sense of purpose may choose entrepreneurship over salaried work to achieve their life goals. Furthermore, Akhtar et al. (2020) found that NA influences self-efficacy. People with high NA have more confidence in their abilities and are more resilient in facing adversity. In addition, they are driven to overcome obstacles and achieve success. Based on the above discussion, we proposed the hypotheses:

*H3:* NA positively and significantly influences (a) EA and (b) PBC among Hong Kong youths.

### Locus of control

Rotter (1966) Locus of control (LOC) is classed as an internal locus of control and an external locus of control. Specifically, an internal locus of control (ILOC) refers to a person’s belief that behaviors control events. In contrast, an external locus of control refers to a person’s belief that circumstances can influence their life and fate or luck are beyond their control (Koh, 1996). Entrepreneurs are often assumed to have a high ILOC, a valuable statistic for separating successful from unsuccessful entrepreneurs (Thomas and Mueller, 2000). Several studies have verified that ILOC influences the intention to start a business (Chaudhary, 2017; Roy et al., 2017). Lüthje and Franke (2003) revealed that ILOC positively and significantly influences attitude. Furthermore, individuals with more internal control points than external control points may have more substantial expectations of their capacity to affect outcomes (Karimi et al., 2017). Based on the discussion, we proposed the hypotheses:

*H4:* ILOC positively and significantly influences (a) EA and (b) PBC among Hong Kong youths.

### Risk-taking propensity

Risk-taking propensity (RTP) is frequently connected with entrepreneurs (Jackson, 1977). Entrepreneurs are more risk-tolerant than others (Thomas and Mueller, 2000) and are considered risk-takers (Taatila, 2010). The previous literature indicated that individuals with greater RTP show greater EI (Karabulut, 2016). A person with a high RTP has higher intentions of starting a venture (Nabi and Liñán, 2013). It is common for entrepreneurs to bear responsibility for the outcomes of risky acts. Starting a new business entails considerable risk and uncertainty, such as the risk of loss of career opportunities and stability, an unbalanced family life, emotional health, and financial problems (Brockhaus Sr, 1980). Thus, those who are more willing to take risks are more prone to choose an entrepreneurial career path (Stewart Jr and Roth, 2001). Furthermore, an individual’s self-efficacy and sense of control might be connected to their perceptions of entrepreneurial risks (Macko and Tyszka, 2009). Specifically, individuals with a higher risk-taking proclivity and a more optimistic risk outlook can expect to be less concerned about an entrepreneurial career, have a stronger sense of control over entrepreneurial outcomes, and place a high value on the likelihood of a successful venture (Zhao et al., 2005). Based on the discussion, we proposed the hypotheses:

*H5:* RTP positively and significantly influences (a) EA and (b) PBC among Hong Kong youths.

### Tolerance for ambiguity

Ambiguity tolerance (TA) requires entrepreneurs to deal with unknown circumstances logically and calmly (Stanley Budner, 1962). Entrepreneurs typically make decisions based on intuition and put significant time and energy into launching a firm with an uncertain outcome (Cromie, 2000; Thomas and Mueller 2000). In this vein, individuals with a high level of TA find ambiguous situations intriguing, exciting, and challenging (Teoh and Foo, 1997), whereas individuals with a low level of TA find uncertain and unstructured environments more unpleasant. Previous research has shown that those with high TA are more entrepreneurial (Koh, 1996). Young entrepreneurs will face many uncertainties, such as a lack of funds and unfavorable products. Still, high TA will motivate them to actively solve these issues, making them feel that starting a business is attractive. Furthermore, an individual’s perceptions of ambiguity may influence their behavioral controls. Since entrepreneurs are always looking for ambiguity and having fun dealing with it (Mitton, 1989) and have the confidence to deal with them, and enable them to believe that they can achieve business success. Based on the discussion, we proposed the hypotheses:

*H6:* TA positively and significantly influences (a) EA and (b) PBC among Hong Kong youths.

### Social networking

According to sociological theory, developing social relationships is essential for pursuing business opportunities (Reynolds, 1992). The opinion that operating a business needs a robust social network is widely accepted in China, particularly among Chinese business people (Lee et al., 2001). According to Burt (1992) social network theory, humans live in a social framework. The major advantage of social relationships is that they provide access to knowledge, counsel, and issue resolution, which supports attitude or behavior change (Hoang and Antoncic, 2003). Furthermore, when people believe they have the necessary resources and opportunities, they should be confident in their capacity to accomplish an activity (Ajzen, 2002). For example, getting a regular flow of funds and information helps them decrease unpredictability (Kristiansen and Ryen, 2002) and find market possibilities (Anderson et al., 2007). Based on the discussion, we proposed the following hypotheses:

*H7:* SNET positively and significantly influences (a) EA and (b) PBC among Hong Kong youths.

### Mediating roles of TPB models’ components

In this study, we proposed some relationships to test based on the Ajzen (1991) model: the effects of EA and PBC on EI and the effects of EI on SP (H1a, H1b, and H1d). In addition, the relationships between six personality traits and EA and PBC have also been proposed to be examined (H2a, H2b, H3a, H3b, H4a, H4b, H5a, H5b, H6a, H6b, H7a, and H7b). Also, prior literature has considered PBC and EA as mediating variables in various relationships connecting individual factors and entrepreneurial results. For instance, Anwar et al. (2021) found EA and entrepreneurial self-efficacy mediate personality traits and EI. Moreover, based on the TPB model, individual attitude is linked to actions *via* behavioral intention (Ajzen, 1991). These research results suggest that there may be a direct or indirect link between one’s attitude and one’s actions. Based on the discussion, we proposed the following hypotheses:

*H8:* EA positively mediates the relationships between (a) NA, (b) CREA, (c) ILOC, (d) RTP, (e) TA, (f) SNET and EI, respectively.

*H9:* PBC positively mediates the relationships between (a) NA, (b) CREA, (c) ILOC, (d) RTP, (e) TA, (f) SNET and EI, respectively.

*H10:* EA and EI positively mediate the relationships between (a) NA, (b) CREA, (c) ILOC, (d) RTP, (e) TA, (f) SNET, and SP, respectively.

*H11:* PBC and EI positively mediate the relationships between (a) NA, (b) CREA, (c) ILOC, (d) RTP, (e) TA, (f) SNET and SP, respectively.

### Control variables

In this study, we used three control variables: (1) entrepreneurship education (EEDU), (2) prior entrepreneurial experience (PEEXP), and (3) work experience (WEXP). Accordingly, Bae et al. (2014) revealed that entrepreneurial education significantly influences EI. Also, Khuong and An (2016) found prior entrepreneurial experience positively influences EI. Zapkau et al. (2015) revealed work experience influences EI. Based on the discussion, we proposed the following hypotheses:

*H12:* (a) EEDU, (b) PEEXP, (c) WEXP positively and significantly influences SP among Hong Kong youths.

### The research model

**Figure 1:**
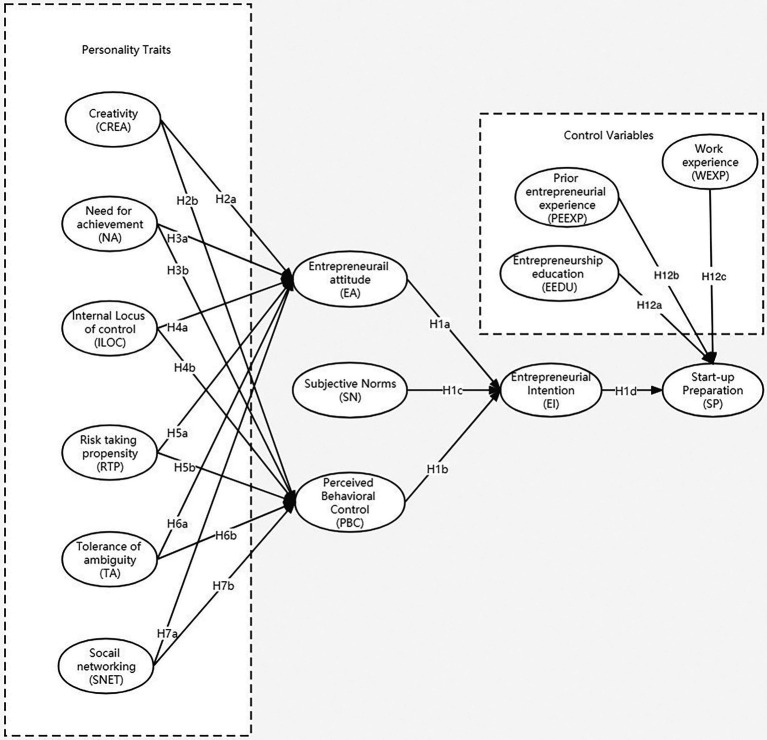
Research model.

Our model was developed based on the integration of (Ajzen, 1991) theory of planned behavior model (TPB) and Schultz (1961) and Becker (2009) human capital theory. A theoretical model with hypotheses was formulated (See Figure 1).

## Materials and methods

### Measures

All measurement scales used in this study were adopted or revised from and validated in prior studies. All the measurement items were measured on a five-point scale, ranging from 1 = strongly disagree and 5 = strongly agree (See Table 2).

**Table 2:** Questionnaire.

Entrepreneurial attitude	
EA1	Being an entrepreneur has more benefits than drawbacks for me
EA2	I am interested in pursuing a career as an entrepreneur
EA3	If I had the opportunity and resources, Id like to start a business
EA4	Being an entrepreneur would provide me with a lot of satisfaction
EA5	Of all the options available to me, I would prefer to be an entrepreneur
Subjective norm	
SN1	If I start my own business, my parents would be supportive
SN2	If I start my own business, my closest friends would be supportive
SN3	If I start my own business, my colleagues or class-mates would be supportive
Perceived behavioral control	
PBC1	I can control the creation process of a new business
PBC2	If I tried to start a business, I would have a high probability of success
PBC3	Starting a business and keeping it functional would be easy for me
PBC4	I know the necessary practical details to start a business
PCB5	I know how to develop an entrepreneurial project
Entrepreneurial intention	
EI1	I am ready to do anything to be an entrepreneur
EI2	My professional goal is to become an entrepreneur
EI3	I will make every effort to start and run my own firm
EI4	I am determined to create a firm in the future
EI5	I have very seriously thought of starting a firm
EI6	I have the firm intention to start a firm some day
Start-up preparation	
SP1	I have prepared a business plan
SP2	I have done market research
SP3	I have formed a start-up team
SP4	I have gathered the information regarding funding support
SP5	I have gathered the information regarding administrative formalities for company registration
Creativity	
CREA1	I frequently surprised people with my novel ideas
CREA2	I find it easy to come up with new, wild or even crazy ideas
CREA3	I prefer a job that requires me to think creatively
CREA4	I prefer to try different ways of doing a similar thing
CREA5	I consider myself as a creative person
Need for achievement	
NA1	I desire and pursue success
NA2	I enjoy situations that, I can make use of my abilities
NA3	I am constantly striving to improve my work performance
NA4	It is important for me to do the best job possible
NA5	I enjoy completing tasks
Internal locus of control	
ILOC1	To a greater extent, I control my own life
ILOC2	My life is determine by my actions
ILOC3	The success of my life is heavily reliant on my ability
ILOC4	Success is usually the result of diligence and hard work
ILOC5	I mostly can control what will happen in my life
Risk taking propensity	
RTP1	I am willing to take risks for high returns
RTP2	I am ready to take risks
RTP3	I take chances regardless of the risks
RTP4	I prefer a business that offers high returns with high risks over a secured job with steady salary
RTP5	I do not fear moving into a new undertaking I know nothing about
Tolerance for ambiguity	
TA1	I can accept unstable work
TA2	In unclear situations, I like to make decisions and take the “lead”
TA3	I enjoy a job without clear instructions
TA4	I enjoy working in unstructured situations
TA5	I can deal with unexpected situations
Social networking	
SNET1	I try to meet people who may be important to me
SNET2	I enjoy establishing social networking
SNET3	I like to talk to people who I do not know yet
SNET4	I enjoy maintaining social networking
SNET5	I enjoy making friends

### Entrepreneurial attitude, subjective norms, perceived behavioral controls, and entrepreneurial intention

The measurement scales of entrepreneurial attitude (EA), subjective norms (SN), perceived behavioral controls (PBC), and entrepreneurial intention (EI) were adopted from Liñán and Chen (2009). Specifically, a sample item of entrepreneurial attitude is “Being an entrepreneur has more benefits than drawbacks for me.” Also, a sample item of subjective norms is “If I start my own business, my parents would be supportive.” and a sample item of perceived behavioral controls (PBC) is “I can control the creation process of a new business.” Finally, a sample item of entrepreneurial intention is “My professional goal is to become an entrepreneur.”

### Start-up preparation

The scales of start-up preparation (SP) were adopted from Mamun et al. (2017) with modifications. The revised items of start-up preparation include “I have prepared a business plan,” “I have done market research,” “I have formed a start-up team,” “I have gathered the information regarding funding support,” and “I have gathered the information regarding administrative formalities for company registration.”

### Creativity, need for achievement, internal locus of control, risk-taking propensity, tolerance of ambiguity, and social networking

The measurement items of the six personality traits were adopted from Taormina and Lao (2007), Karabulut (2016), and Mahmood et al. (2019). A sample item of creativity (CREA) is “I frequently surprised people with my novel ideas.” A sample item of the need for achievement (NA) is “I desire and pursue success.” A sample item of internal locus of control (ILOC) is “To a greater extent, I control my own life.” A sample item of risk-taking propensity (RTP) is “I am willing to take risks for high returns.” A sample item of tolerance of ambiguity (TA) is “I can deal with unexpected situations.” A sample item of social networking (SNET) is “I try to meet people who may be important to me.”

### Entrepreneurship education, prior entrepreneurship experience, and work experience

We collected data on entrepreneurship education by asking respondents to answer “yes” or “no.” Also, we collected data on years of “prior entrepreneurship experience” and “work experience” by asking respondents to fill in the numbers in the blanks. In the analysis, ‘prior entrepreneurship experience’ and ‘work experience’ were treated as continuous value. Regarding entrepreneurship education, a dummy variable was adopted (“1” if young people have attended any innovation and entrepreneurship course and/or training and “0” if they have not).

### Pilot testing

Since we adopted the measurement scales from previous studies, we conducted pilot testing before the formal data collection. We shared the link on social platforms and provide a blank for comments about the filling time and content. Finally, we received 23 questionnaires. The pilot test of the survey showed that the filling time was acceptable and most of the expressions of items are understandable except for some words. Accordingly, we revised the confused words and updated the questionnaire.

### Participants and procedure

#### Sample size

Hair et al. (2011) suggest that the sample size of PLS-SEM should be equal to the larger of: (1) 10 times the largest number of formative indicators used to measure a single construct, or (2) 10 times the largest number of structural paths directed at a particular construct in the structural model. Since we only have reflective constructs, the minimum number of samples should follow rule (2). More specifically, the number of paths directed at either entrepreneurial attitude or perceived behavioral controls is six, and therefore, this study should have at least 60 questionnaires.

The questionnaire was created and demonstrated in the *Google Form* using English. Then, a simple random sampling was used to collect data in multiple channels. From January to March 2022, the questionnaire link was shared on social networking platforms and our seminars and the help of a consulting company. To gain the consent of the respondents, a data collection statement before the survey will be ticked. First, questionnaire participants were informed that they could withdraw at any time during the filling process. Second, participants were also informed that the questionnaire is anonymous without any personal information collected. Finally, because our research population is Hong Kong youths aged from 18 to 40, the questionnaires from the respondents outside Hong Kong and those aged over 40 were excluded. Finally, 230 sets of valid questionnaires were used for further analysis. The sample description of the respondents’ gender, age, major of study, educational level, and monthly household income was shown in Table 3.

**Table 3:** Sample description of respondents (*n* = 230).

Categories		Frequency	Percentage
Gender	Male	81	35.2%
	Female	149	64.7%
	Total	230	100%
Age	18–24	167	72.6%
	25–30	43	18.7%
	31–40	20	8.7%
Education	High School or lower	14	6.1%
	Vocational degree	20	8.7%
	Bachelor’s degree	159	69.1%
	Master’s degree	36	15.7%
	Doctoral degree	1	0.4%
	Total	230	100%
Major	Engineering	55	23.9%
	Management, Business and Economics	42	18.3%
	Medicine or Biology	21	9.1%
	Science	16	7.0%
	Social science	43	18.7%
	Other	53	23.0%
	Total	230	100%
Monthly household income	Low income	91	39.6%
	Middle income	136	59.1%
	High income	3	1.3%
	Total	230	100%

### Analysis method and software

To test the proposed model, we adopt partial least squares structural equation modeling (PLS-SEM) by using Smart PLS software (version 3.3.3) due to the exploratory purposes and a large number of constructs and indicators (Hair et al., 2011). The analysis process includes a measurement model and a structural model.

### Assessment of measurement model—confirmatory factor analysis

There are two kinds of constructs: formative and reflective. Based on the definition of reflective constructs, the items of one reflective construct should be based on the same or similar content (Urbach and Ahlemann, 2010). Accordingly, all the constructs are treated as reflective. Then, we followed the guidelines for evaluating reflective constructs, consisting of internal consistency reliability, indicator reliability, convergent validity, and discriminant validity (Hair et al., 2011). As Table 4 shows, first, the values of all constructs’ Cronbach’s are greater than 0.7 (Cronbach, 1951) and the values of all constructs’ composite reliability (CR) are greater than 0.7 (Nunnally, 1994), indicating that all the constructs have good internal consistency reliability. Second, indicator loading of RTP3 (0.559) and ILOC4 (0.617) less than 0.7 are eliminated from the measurement model (Chin, 1998) and nearly all indicators indicate good indicator reliability. Third, the average variance extracted (AVE) values of all constructs are larger than 0.5, indicating each construct possesses a high degree of convergent validity (Fornell and Larcker, 1981). Fourth, Table 5 shows that all cross-loadings are smaller than factors loading (Chin, 1998), meeting the requirements. Fifth, Table 6 shows that the square root of each construct’s AVE is greater than the correlation of the construct to other latent variables (Fornell and Larcker, 1981), meeting the requirements. Finally, Table 7 shows that the Heterotrait–Monotrait Ratio (HTMT) values are all smaller than 0.85 (Henseler et al., 2015), meeting the requirements. These three tables indicate all constructs possess a high degree of convergent validity.

**Table 4:** Construct reliability and validity.

	Factor loading	Cronbach’s alpha	Composite reliability (CR)	Average variance extracted (AVE)
EA		0.895	0.923	0.707
EA1	0.731			
EA2	0.886			
EA3	0.83			
EA4	0.86			
EA5	0.886			
SN		0.759	0.861	0.675
SN1	0.72			
SN2	0.853			
SN3	0.882			
PBC		0.861	0.9	0.644
PBC1	0.751			
PBC2	0.854			
PBC3	0.834			
PBC4	0.75			
PBC5	0.816			
EI		0.939	0.952	0.769
EI1	0.731			
EI2	0.886			
EI3	0.83			
EI4	0.86			
EI5	0.886			
EI6	0.731			
SP		0.951	0.962	0.836
SP1	0.903			
SP2	0.902			
SP3	0.934			
SP4	0.898			
SP5	0.934			
CREA		0.925	0.943	0.769
CREA1	0.878			
CREA2	0.875			
CREA3	0.905			
CREA4	0.837			
CREA5	0.887			
ILOC		0.778	0.852	0.592
ILOC1	0.816			
ILOC2	0.71			
ILOC3	0.744			
ILOC4	0.617(removed)			
ILOC5	0.776			
NA		0.885	0.915	0.683
NA1	0.832			
NA2	0.827			
NA3	0.862			
NA4	0.795			
NA5	0.815			
RTP		0.869	0.911	0.72
RTP1	0.859			
RTP2	0.867			
RTP3	0.559(removed)			
RTP4	0.878			
RTP5	0.758			
TA		0.895	0.922	0.703
TA1	0.794			
TA2	0.854			
TA3	0.863			
TA4	0.814			
TA5	0.864			
SNET		0.906	0.93	0.727
SNET1	0.834			
SNET2	0.892			
SNET3	0.876			
SNET4	0.836			
SNET5	0.822			

**Table 5:** Cross loadings.

	EA	SN	PBC	EI	SP	CREA	ILOC	NA	RTP	TA	SNET
EA1	**0.732**	0.285	0.317	0.451	0.208	0.238	0.197	0.362	0.364	0.298	0.257
EA2	**0.886**	0.302	0.489	0.722	0.333	0.476	0.333	0.34	0.565	0.552	0.337
EA3	**0.83**	0.365	0.455	0.58	0.219	0.402	0.274	0.341	0.476	0.455	0.314
EA4	**0.86**	0.315	0.458	0.577	0.26	0.324	0.278	0.367	0.456	0.367	0.32
EA5	**0.886**	0.264	0.531	0.69	0.402	0.374	0.293	0.284	0.523	0.452	0.309
SN1	0.273	**0.72**	0.198	0.203	0.098	0.294	0.211	0.233	0.204	0.195	0.117
SN2	0.241	**0.853**	0.101	0.153	0.02	0.174	0.181	0.31	0.222	0.174	0.045
SN3	0.348	**0.882**	0.276	0.269	0.163	0.239	0.262	0.309	0.277	0.294	0.191
PBC1	0.432	0.173	**0.751**	0.473	0.329	0.384	0.346	0.318	0.45	0.433	0.352
PBC2	0.53	0.173	**0.855**	0.603	0.474	0.443	0.322	0.257	0.563	0.439	0.319
PBC3	0.498	0.276	**0.834**	0.587	0.458	0.377	0.333	0.18	0.47	0.356	0.303
PBC4	0.242	0.206	**0.75**	0.402	0.438	0.227	0.194	0.101	0.352	0.206	0.288
PBC5	0.423	0.19	**0.816**	0.593	0.574	0.402	0.221	0.212	0.542	0.468	0.375
EI1	0.597	0.171	0.668	**0.837**	0.466	0.459	0.289	0.215	0.577	0.462	0.37
EI2	0.686	0.168	0.58	**0.902**	0.491	0.493	0.272	0.24	0.596	0.524	0.391
EI3	0.596	0.271	0.545	**0.806**	0.348	0.429	0.323	0.288	0.49	0.413	0.411
EI4	0.687	0.265	0.632	**0.939**	0.577	0.572	0.327	0.271	0.619	0.538	0.446
EI5	0.593	0.251	0.536	**0.872**	0.519	0.529	0.298	0.32	0.543	0.494	0.417
EI6	0.674	0.284	0.577	**0.898**	0.518	0.503	0.275	0.281	0.591	0.493	0.395
SP1	0.34	0.128	0.544	0.539	**0.903**	0.375	0.211	0.151	0.457	0.422	0.271
SP2	0.317	0.085	0.545	0.509	**0.902**	0.388	0.206	0.154	0.45	0.423	0.28
SP3	0.329	0.134	0.523	0.529	**0.934**	0.349	0.217	0.147	0.44	0.434	0.274
SP4	0.282	0.097	0.46	0.465	**0.898**	0.312	0.219	0.116	0.428	0.38	0.278
SP5	0.309	0.148	0.53	0.508	**0.934**	0.345	0.226	0.167	0.45	0.416	0.295
CREA1	0.357	0.294	0.439	0.49	0.358	**0.878**	0.318	0.349	0.483	0.525	0.386
CREA2	0.363	0.217	0.391	0.512	0.34	**0.875**	0.241	0.307	0.476	0.528	0.388
CREA3	0.465	0.302	0.41	0.56	0.357	**0.905**	0.274	0.42	0.492	0.552	0.399
CREA4	0.378	0.272	0.341	0.441	0.269	**0.837**	0.271	0.471	0.424	0.534	0.45
CREA5	0.365	0.2	0.457	0.487	0.368	**0.887**	0.331	0.419	0.491	0.545	0.409
ILOC1	0.308	0.215	0.324	0.321	0.228	0.356	**0.847**	0.457	0.302	0.363	0.388
ILOC2	0.122	0.31	0.16	0.148	0.124	0.263	**0.698**	0.456	0.17	0.196	0.196
ILOC3	0.225	0.249	0.241	0.224	0.155	0.209	**0.741**	0.427	0.236	0.27	0.257
ILOC5	0.299	0.144	0.314	0.29	0.19	0.187	**0.784**	0.34	0.405	0.379	0.317
NA1	0.409	0.346	0.279	0.311	0.165	0.362	0.48	**0.832**	0.343	0.371	0.405
NA2	0.282	0.336	0.191	0.213	0.138	0.406	0.374	**0.827**	0.281	0.343	0.279
NA3	0.343	0.235	0.266	0.297	0.136	0.445	0.432	**0.862**	0.372	0.394	0.413
NA4	0.283	0.279	0.174	0.203	0.124	0.268	0.442	**0.795**	0.237	0.276	0.288
NA5	0.287	0.224	0.172	0.2	0.089	0.356	0.448	**0.815**	0.246	0.341	0.34
RTP1	0.469	0.31	0.54	0.545	0.392	0.512	0.428	0.347	**0.857**	0.587	0.384
RTP2	0.486	0.281	0.54	0.603	0.441	0.515	0.349	0.328	**0.887**	0.623	0.427
RTP4	0.577	0.222	0.494	0.586	0.443	0.396	0.285	0.279	**0.876**	0.664	0.422
RTP5	0.409	0.169	0.468	0.468	0.373	0.413	0.232	0.293	**0.769**	0.63	0.354
TA1	0.402	0.288	0.373	0.45	0.391	0.456	0.193	0.317	0.701	**0.794**	0.385
TA2	0.484	0.25	0.48	0.556	0.424	0.562	0.42	0.382	0.644	**0.854**	0.467
TA3	0.409	0.171	0.421	0.438	0.41	0.462	0.369	0.334	0.627	**0.863**	0.444
TA4	0.402	0.235	0.285	0.374	0.287	0.473	0.361	0.329	0.513	**0.814**	0.429
TA5	0.452	0.245	0.443	0.492	0.378	0.592	0.369	0.396	0.596	**0.864**	0.465
SNET1	0.306	0.19	0.329	0.367	0.221	0.445	0.366	0.471	0.404	0.446	**0.834**
SNET2	0.312	0.106	0.355	0.377	0.266	0.354	0.368	0.326	0.346	0.42	**0.892**
SNET3	0.368	0.139	0.432	0.491	0.363	0.444	0.351	0.343	0.511	0.579	**0.876**
SNET4	0.291	0.119	0.316	0.353	0.236	0.335	0.318	0.336	0.381	0.389	**0.836**
SNET5	0.271	0.125	0.281	0.351	0.18	0.381	0.268	0.351	0.325	0.36	**0.822**

Values with bold font show indicator loadings of each construct.

**Table 6:** Fornell–Lacker criterion.

	EA	SN	PBC	EI	SP	CREA	ILOC	NA	RTP	TA	SNET
EA	**0.841**										
SN	0.362	**0.821**									
PBC	0.543	0.291	**0.802**								
EI	0.73	0.267	0.673	**0.877**							
SP	0.346	0.13	0.57	0.559	**0.915**						
CREA	0.441	0.293	0.467	0.569	0.388	**0.877**					
ILOC	0.332	0.274	0.356	0.338	0.236	0.328	**0.769**				
NA	0.398	0.346	0.271	0.305	0.161	0.448	0.53	**0.826**			
RTP	0.575	0.253	0.602	0.652	0.487	0.541	0.383	0.367	**0.849**		
TA	0.516	0.283	0.485	0.558	0.455	0.612	0.413	0.422	0.737	**0.838**	
SNET	0.367	0.16	0.408	0.461	0.305	0.462	0.395	0.426	0.469	0.524	**0.853**

Values with bold font show the square root of AVE.

**Table 7:** Heterotrait-monotrait ratio (HTMT).

	EA	SN	PBC	EI	SP	CREA	ILOC	NA	RTP	TA	SNET
EA											
SN	0.427										
PBC	0.596	0.29									
EI	0.784	0.302	0.737								
SP	0.366	0.136	0.625	0.586							
CREA	0.473	0.343	0.51	0.608	0.411						
ILOC	0.363	0.376	0.406	0.373	0.261	0.386					
NA	0.442	0.419	0.295	0.327	0.171	0.491	0.65				
RTP	0.641	0.349	0.685	0.717	0.534	0.603	0.435	0.409			
TA	0.562	0.327	0.531	0.6	0.488	0.668	0.462	0.466	0.835		
SNET	0.402	0.179	0.455	0.494	0.32	0.504	0.442	0.468	0.519	0.569	

### Common method bias

Harman’s single-factor test was widely used to test common method biases (Fuller et al., 2016). Results of Harman’s single-factor test showed that the most covariance explained by one factor is 35.040%, smaller than 50%, indicating that common method biases are not a likely contaminant of our results (Podsakoff et al., 2003).

## Results

We follow the rules of evaluating the structural model (Urbach and Ahlemann, 2010; See Table 8). First, we need to assess the coefficient of determination (*R*^2^). The value of *R*^2^ indicates the amount of independent variables explained dependent variables (Chin, 1998). *R*^2^ values of.190, 0.333, and.670 suggest that endogenous latent variables in the structural model are weak, moderate, and substantial, respectively (Chin 1998). Regarding *R*^2^, entrepreneurial intention (EI) (*R*^2^ = 0.642), entrepreneurial attitude (EA) (*R*^2^ = 0.385), and perceived behavioral control (PBC) (*R*^2^ = 0.414) are considered moderate and start-up preparation (SP) (*R*^2^ = 0.313) is considered as weak. Second, to assess the path coefficients’ significance in structural path analysis, the signs and statistical significance of path coefficients are used to test the proposed hypotheses through 5,000 bootstrap samples (Hair et al., 2011) and path coefficients should be significant at least at the.050 level (*t*-value > 1.96).

**Table 8:** Path coefficients.

Hypotheses	Beta	Mean	SD	t-value	p-value	f^2^	S.g.	Decision
H12a: EEDU → SP	0.242	0.239	0.066	3.691	0		***	Supported
H12b: PEEXP → SP	0.175	0.178	0.069	2.558	0.011		*	Supported
H12c: WEXP → SP	−0.063	−0.059	0.061	1.029	0.303		n.s.	Not supported
EA (*R*^2^ = 0.385; *Q*^2^ = 0.244)								
H2a: CREA → EA	0.091	0.092	0.09	1.016	0.31	0.008	n.s.	Not supported
H3a: NA → EA	0.154	0.155	0.076	2.023	0.043	0.024	*	Supported
H4a: ILOC → EA	0.028	0.037	0.08	0.353	0.724	0.001	n.s.	Not supported
H5a: RTP → EA	0.381	0.376	0.092	4.13	0	0.103	***	supported
H6a: TA → EA	0.091	0.09	0.101	0.902	0.367	0.005	n.s.	Not supported
H7a: SNET → EA	0.022	0.023	0.07	0.309	0.757	0	n.s.	Not supported
PBC (*R*^2^ = 0.414; *Q*^2^ = 0.255)								
H2b: CREA → PBC	0.187	0.187	0.07	2.685	0.007	0.033	**	Supported
H3b: NA → PBC	−0.082	−0.08	0.067	1.227	0.22	0.007	n.s.	Not supported
H4b: ILOC → PBC	0.137	0.136	0.058	2.356	0.019	0.021	*	Supported
H5b: RTP → PBC	0.464	0.462	0.069	6.695	0	0.159	***	Supported
H6b: TA → PBC	−0.052	−0.052	0.085	0.608	0.543	0.002	n.s.	Not supported
H7b: SNET → PBC	0.112	0.117	0.061	1.853	0.064	0.014	n.s.	Not supported
EI (*R*^2^ = 0.642; *Q*^2^ = 0.483)								
H1a: EA → EI	0.524	0.522	0.052	10.053	0	0.5	***	Supported
H1b: PBC → EI	0.394	0.396	0.052	7.656	0	0.305	***	Supported
H1c: SN → EI	−0.022	−0.017	0.044	0.504	0.614	0.001	n.s.	Not supported
SP (*R*^2^ = 0.313; *Q*^2^ = 0.328)								
H1d: EI → SP	0.478	0.474	0.05	9.585	0	0.455	***	Supported

* *p* < 0.05;

** *p* < 0.01;

*** *p* < 0.001.

### Direct effects

**Figure 2:**
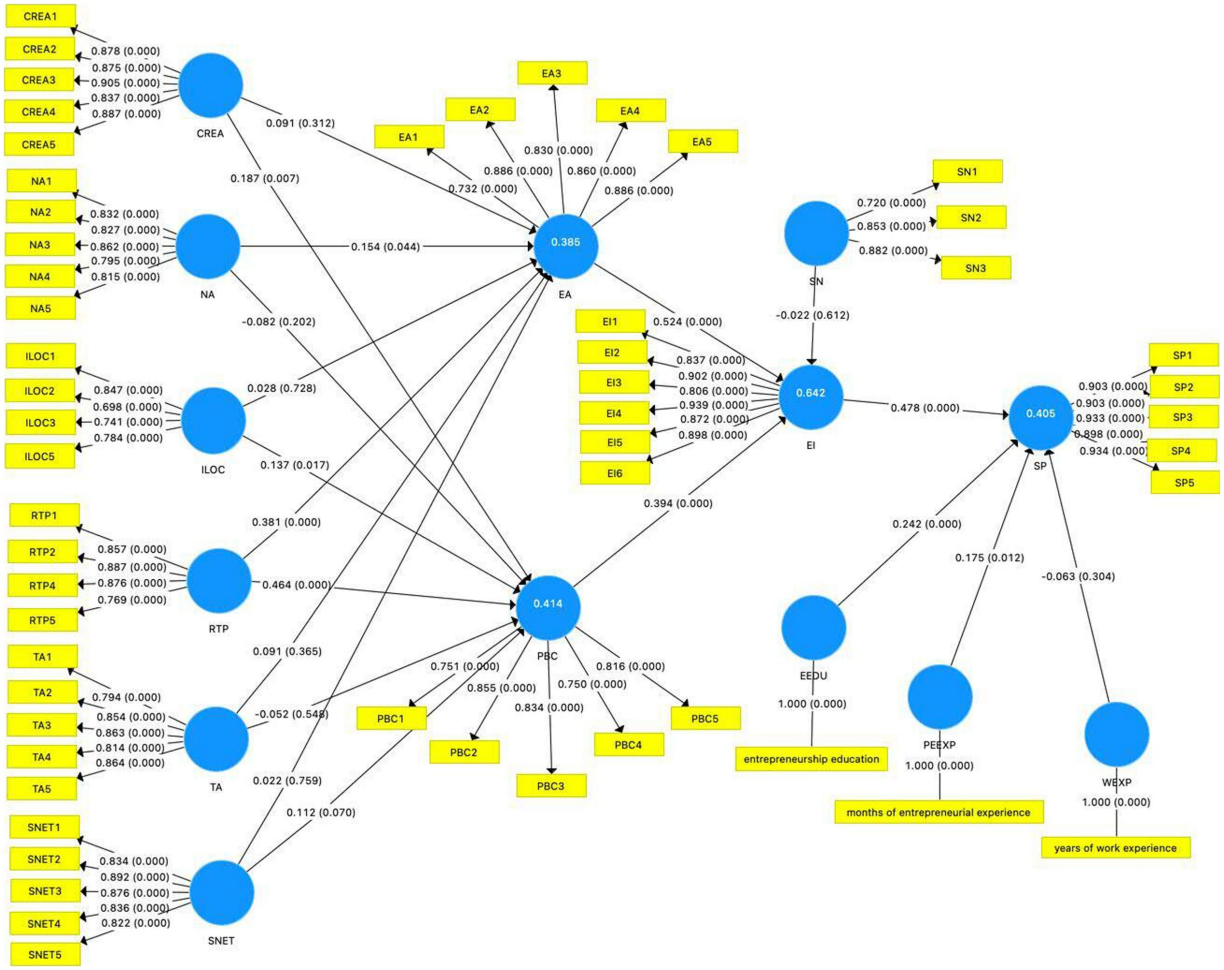
Results of the model.

Some hypotheses are supported by the bootstrapping result (See Figure 2). Among the relationships among the theory of the planned behavior model, our study revealed that entrepreneurial attitude is positive and significantly related to entrepreneurial intention (*β* = 0.524; *value of p* = 0.000), then H1a is supported. Also, perceived behavioral control is positively and significantly related to entrepreneurial intention (*β* = 0.394; *value of p* = 0.000), then H1b is supported. Moreover, entrepreneurial intention is positively and significantly related to start-up preparation, then H1d is supported. However, the subjective norms is non-significantly related with entrepreneurial intention (*β* = −0.022; *value of p* = 0.614), then H1c is not supported.

Furthermore, among the relationships between six personality traits and entrepreneurial attitude and perceived behavioral control, we revealed that creativity is positively and significantly related with perceived behavioral control (*β* = 0.187; *value of p* = 0.007) but non-significantly related with entrepreneurial attitude (*β* = 0.091; *value of p* = 0.31), then H2a is not supported and H2b is supported. Also, the need for achievement is positively and significantly related with entrepreneurial attitude (*β* = 0.154; *value of p* = 0.043) but non-significantly related with perceived behavioral control (*β* = −0.082 *value of p* = 0.22), then H3a is supported and H3b is not supported. Moreover, internal locus of control is positively and significantly related with perceived behavioral control (*β* = 0.137; *value of p* = 0.019) but non-significantly related with entrepreneurial attitude (*β* = 0.028; *value of p* = 0.724), then H4a is not supported and H4b is supported. However, we found risk-taking propensity positively and significantly related with both entrepreneurial attitude (*β* = 0.381; *value of p* = 0.000) and perceived behavioral control (*β* = 0.464; *value of p* = 0.000), then H5a and H5b are supported. Further, tolerance for ambiguity is non-significantly related with both entrepreneurial attitude (*β* = 0.091; *value of p* = 0.367) and perceived behavioral control (*β* = −0.052; *value of p* = 0.543), then H6a and H6b are not supported. Similarly, social networking is non-significantly related with both entrepreneurial attitude (*β* = 0.022; *value of p* = 0.757) and perceived behavioral control (*β* = 0.112; *value of p* = 0.064), then H7a and H7b are not supported.

Third, the value of predictive relevance (Q^2^) larger than zero indicates the model’s predictive relevance for latent variables (Geisser, 1975). The values of predictive relevance of entrepreneurial intention (EI; *Q*^2^ = 0.483), entrepreneurial attitude (EA; *Q*^2^ = 0.244), perceived behavioral control (PBC, *Q*^2^ = 0.255), and start-up preparation (SP; *Q*^2^ = 0.328) are larger than the threshold.

Fourth, Cohen (1988) effect sizes (*f*^2^) showed the effects of latent independent variables on latent dependent variables larger than 0.020, 0.150, and 0.350, indicating low, medium, and large effects, respectively (Chin, 1998). Therefore, the effect sizes of EA ->EI and EI ->SP are large and the effects of RTP ->PBC and PBC ->EI are medium, attaining values between 0.150 and 0.350. Also, the effect sizes of RTP ->EA, NA ->EA, CREA ->PBC, and ILOC ->PBC are low. However, the effects of CREA ->EA, ILOC ->EA, TA ->EA, SNET ->EA, NA ->PBC, TA->PBC, SNET ->PBC, and SN ->EI are none. The results of the model are shown in Figure 2.

### Indirect effects

Regarding the effects of single mediator variable, entrepreneurial attitude (See Table 9), we found that entrepreneurial attitude (EA) positively mediates the relationship between need for achievement (NA) and entrepreneurial intention (EI; *β* = 0.081; *value of p* = 0.04). Then, H8a is supported. Similarly, entrepreneurial attitude (EA) positively mediates the relationship between risk-taking propensity (RTP) and entrepreneurial intention (EI; *β* = 0.2; *value of p* = 0.000). Then, H8d is supported. The mediating relationships of entrepreneurial attitude between other personality traits (creativity, internal locus of control, tolerance of ambiguity, and social networking) and entrepreneurial intention are non-significant, then H8b, H8c, H8e, and H8f are not supported.

**Table 9:** Special indirect effects.

Hypotheses	Beta	Mean	SD	t-value	p-value	S.g.	Decision
H8a: NA → EA → EI	0.081	0.08	0.039	2.052	0.04	*	Supported
H8b: CREA → EA → EI	0.048	0.049	0.049	0.976	0.329	n.s.	Not supported
H8c: ILOC → EA → EI	0.015	0.019	0.043	0.35	0.726	n.s.	Not supported
H8d: RTP → EA → EI	0.2	0.198	0.052	3.832	0	***	Supported
H8e: TA → EA → EI	0.048	0.046	0.054	0.882	0.378	n.s.	Not supported
H8f: SNET → EA → EI	0.011	0.013	0.036	0.311	0.756	n.s.	Not supported
H9a: NA → PBC → EI	−0.032	−0.032	0.027	1.207	0.227	n.s.	Not supported
H9b: CREA → PBC → EI	0.074	0.074	0.031	2.397	0.017	*	Supported
H9c: ILOC → PBC → EI	0.054	0.055	0.024	2.213	0.027	*	Supported
H9d: RTP → PBC → EI	0.183	0.184	0.037	4.992	0	***	Supported
H9e:TA → PBC → EI	−0.02	−0.02	0.034	0.597	0.55	n.s.	Not supported
H9f: SNET → PBC → EI	0.044	0.047	0.026	1.718	0.086	n.s.	Not supported
H10a: NA → EA → EI → SP	0.039	0.038	0.019	2.034	0.042	*	Supported
H10b: CREA → EA → EI → SP	0.023	0.023	0.023	0.974	0.33	n.s.	Not supported
H10c: ILOC → EA → EI → SP	0.007	0.009	0.02	0.353	0.724	n.s.	Not supported
H10d: RTP → EA → EI → SP	0.095	0.094	0.026	3.674	0	***	Supported
H10e: TA → EA → EI → SP	0.023	0.022	0.026	0.875	0.382	n.s.	Not supported
H10f: SNET → EA → EI → SP	0.005	0.006	0.017	0.311	0.755	n.s.	Not supported
H11a: NA → PBC → EI → SP	−0.015	−0.015	0.013	1.173	0.241	n.s.	Not supported
H11b: CREA → PBC → EI → SP	0.035	0.035	0.016	2.252	0.024	*	Supported
H11c: ILOC → PBC → EI → SP	0.026	0.026	0.012	2.086	0.037	*	Supported
H11d: RTP → PBC → EI → SP	0.087	0.087	0.02	4.282	0	***	Supported
H11e: TA → PBC → EI → SP	−0.01	−0.009	0.016	0.594	0.553	n.s.	Not supported
H11f: SNET → PBC → EI → SP	0.021	0.022	0.013	1.631	0.103	n.s.	Not supported

* *p* < 0.05.

** *p* < 0.01.

*** *p* < 0.001.

Further, regarding the effects of the single mediator variable (See Table 9), perceived behavioral control (PBC) positively mediates between creativity (CREA) and entrepreneurial intention (EI; *β* = 0.074; *value of p* = 0.017), then H9b is supported. Also, perceived behavioral control (PBC) positively mediates between internal locus of control (ILOC) and entrepreneurial intention (EI; *β* = 0.054; *value of p* = 0.027), then H9c is supported. Similarly, perceived behavioral control (PBC) positively mediates between risk-taking propensity (RTP) and entrepreneurial intention (EI; *β* = 0.183; *value of p* = 0.000), then H9d is supported. However, the mediating relationships of perceived behavioral control between other personality traits (need for achievement, tolerance of ambiguity, and social networking) and entrepreneurial intention are non-significant, then H9a, H9e, and H9f are not supported.

Finally, regarding the effects of the two mediator variables, the results are similar to those found in the single mediator variable. Specifically, entrepreneurial attitude and entrepreneurial intention positively mediate the need for achievement (NA) and start-up preparation (SP) (*β* = 0.039; *value of p* = 0.042). Also, entrepreneurial attitude and entrepreneurial intention positively mediate risk-taking propensity (RTP) and start-up preparation (SP) (*β* = 0.095; *value of p* = 0.000). Then, H10a and H10d are supported while H10b, H10c, H10e, and H10f are not supported.

We also found that perceived behavioral control (PBC) and entrepreneurial intention (EI) positively mediate the relationships only between creativity (CREA) (*β* = 0.035; *value of p* = 0.024), internal locus of control (ILOC) (*β* = 0.026; *value of p* = 0.037), risk-taking propensity (RTP) (*β* = 0.087; *value of p* = 0.000), and start-up preparation (SP), respectively. Then H11b, H11c, and H11d are supported, while H11a, H11e, and H11f are not supported.

## Discussion

There are some key findings, as shown below.

The first finding was among three predictors of entrepreneurial intention (EI). We found only two antecedents’ significant effects (entrepreneurial attitude (EA), perceived behavioral control (PBC)) on EI and the non-significant effect of the subjective norms (SN) on EI. It means that the support from families, friends, and colleagues or classmates does not motivate Hong Kong people to start a business. This result is consistent with the findings of earlier research (Liñán and Chen, 2009; Moriano et al., 2012). Moreover, the EA has a large effect, whereas PBC has a medium effect on EI. It means that changing Hong Kong people’s entrepreneurial attitude would be effective in enhancing their entrepreneurial intention.

The second finding was that this model describes 64.2% of the variance in EI, which is higher than what has been observed in prior research on EI (Krueger et al., 2000; Liñán and Chen, 2009). For example, Krueger et al. (2000) revealed that the TPB accounts for 35% of the variation in EI among university business students. For Spanish students, Liñán and Chen (2009) suggested the TPB model explained 58% of the variation in EI. Moreover, EI explained 40.5% of the variance in start-up preparation (SP). This finding exceeds that of Kautonen et al. (2013), who found that intention explained 31% of entrepreneurial behavior. This means personality traits can contribute to the start-up preparation.

The third finding was the effects of six personality traits on entrepreneurial attitude (EA) and perceived behavioral control (PBC). Specifically, we found the positive and significant effects of the need for achievement (NA) on EA. This result is consistent with the findings of Karabulut (2016) discovered that people with a strong demand for accomplishment aspire to show themselves as successful entrepreneurs. This study also revealed that risk-taking propensity (RTP) positively and significantly influenced entrepreneurial attitude and RTP (*β =* 0.381) is more influential to entrepreneurial attitude compared with need for achievement (*β =* 0.154), which is in line with the importance of risk-taking propensity on EI revealed by previous studies (Nabi and Liñán, 2013; Karabulut, 2016). However, the non-significant effect of creativity (CREA) on EA is opposite to the finding of Miranda et al. (2017) who revealed that CREA positively influences attitude.

Further, we also revealed the positive and significant effects of creativity (CREA; *β =* 0.187), internal locus of control (ILOC; *β =* 0.137), and risk-taking propensity (RTP; *β =* 0.464) on perceived behavioral control (PBC). Similarly, among these three personality traits, RTP has the greatest influence on PBC. However, the non-significant relationship between the need for achievement and PBC is similar to the finding of Uysal et al. (2021), who revealed need for achievement positively influences self-efficacy. Notably, both tolerance of ambiguity (TA) and social networking (SNET) are non-significant related with EA and PBC, which contradicts the findings of Taormina and Lao (2007), who found the importance of social networking for starting a venture in the Chinese context and the importance of tolerance of ambiguity revealed by Nasip et al. (2017). Generally, most personality traits’ effects on EA and PBC are low, and even none and only RTP positively influence EA (*β* = 0.381) and PBC (*β* = 0.464).

The final findings were that the result of special indirect effects revealed that need for achievement (NA) and risk-taking propensity (RTP) influence the entrepreneurial intention (EI) *via* entrepreneurial attitude (EA), whereas creativity (CREA), internal locus of control (ILOC), and risk-taking propensity (RTP) influence the EI *via* perceived behavioral control (PBC). RTP is also the most important personality trait to indirectly influence EI (*β >* 0.1). Furthermore, NA and RTP influence the start-up preparation (SP) *via* EA and EI, whereas CREA, ILOC, and RTP influence the SP *via* PBC and EI. Although RTP also served as an influential personality trait to have indirect effects on SP, the effects are really small (*β <* 0.1).

Drawing on several control variables used to study EI (Zhao et al., 2005; Karabulut, 2016), we used three control variables from human capital theory to control the effects on SP due to the different individual’s entrepreneurial backgrounds. The finding revealed that only prior entrepreneurial experience and entrepreneurship education positively and significantly influence SP.

There are a few theoretical and practical implications of this research which will be explored below.

### Theoretical implication

The purpose of this study is to explore the mediating effects of the TPB model’s constructs between personality traits, entrepreneurial intention, and start-up preparation. Regarding the TPB model, attitude toward entrepreneurship and perceived behavioral controls positively influence entrepreneurial intention. However, subjective norms are non-significant with entrepreneurial intention. That is to say, the more positive attitude toward starting a business and the more perceived behavioral controls Hong Kong young people have, the higher entrepreneurial intention they will have.

Previous studies have proved that personality traits influence entrepreneurial intention (Gurel et al., 2010; Altinay et al., 2012) and the mediating effects of some factors (e.g., self-efficacy, perceived desirability, and perceived feasibility; Roy et al., 2017; Rosique-Blasco et al., 2018). Except for those widely studied relationships in the TPB model, this study enriches the existing literature about personality traits and the TPB model in the field of entrepreneurship in three aspects. First, the results of creativity, need for achievement, internal locus of control, and risk-taking propensity suggest that personality traits do have direct influences on the TPB model’s constructs. Second, the positive relationship between entrepreneurial intention and start-up preparation highlights the function of entrepreneurial intention in predicting start-up preparation. Third, four personality traits’ indirect effects on entrepreneurial intention and start-up preparation suggest that the TPB model’s construct can mediate the relationship between personality traits and start-up preparation.

Finally, the positive influence of prior entrepreneurial experience and entrepreneurship education elaborates on the effects of human capital theory on young people’s start-up preparation. This enriches current research by adopting the human capital theory. For example, those young people’s entrepreneurship research should consider the control variables’ of human capital theory.

### Practical implication

There are some practical implications of this study. Our study highlighted the importance of creativity, need for achievement, internal locus of control, and risk-taking propensity among Hong Kong youths. These four personality traits not only influence young people’s entrepreneurial attitude and perceived behavioral controls, but also their entrepreneurial intention and start-up preparation. Most importantly, risk-taking propensity has the greatest direct impact on entrepreneurial attitude, and perceived behavioral controls and the greatest indirect impact on entrepreneurial intention and start-up preparation. In this vein, the Hong Kong government should take measures to enhance young people’s personality traits and prioritize improving their risk-taking propensity. For example, the government can provide funding support, enhance young people’s capabilities to handle venture risks as well as provide free consultations.

The analysis results also highlighted the importance of prior entrepreneurial experience and entrepreneurship education. Entrepreneurship education positively influences start-up preparation. Based on this finding, we suggest that the government should expose individuals to more entrepreneurship education. Young people of different ages have different opportunities to receive entrepreneurship education. For example, those people who have left campus have no easy access to entrepreneurship education. In this case, the government should cooperate with higher education institutions to set up social entrepreneurship courses.

In addition, prior entrepreneurial experience positively influences start-up preparation. To enhance their entrepreneurial experience, we suggest the government should give more entrepreneurial practice opportunities for young people. We also suggest under the social entrepreneurship education setting, interactive and action-orientated methods should be introduced such as experiential learning and learning-by-doing activities since those teaching methods are useful for gaining entrepreneurial experience (Jones and Iredale, 2010; Arranz et al., 2017).

## Limitations and further research

This study has three limitations which lead to corresponding further research opportunities. First, although this study moved downstream to study entrepreneurial preparation, we only focused on six personality traits, such as creativity, need for achievement, and risk-taking propensity. There are still other factors that might have indirect effects on start-up preparation, such as family backgrounds, role models, and institutional environments. Further research can study the indirect effects on start-up preparation by considering these points.

Second, due to time constraints, our study was cross-sectional. The stability of the link relationship between entrepreneurial intention and start-up preparation remains unclear. Furthermore, although we have studied entrepreneurial intention and start-up preparation, the link between preparation and actual action to start a business is unknown. Fayolle and Liñán (2014) emphasized that further research should be placed on the relationships between the start-up process (Fayolle and Liñán, 2014), so we suggested that further research can conduct longitudinal studies on entrepreneurial intentions and start-up preparation to test the stability and the examination of the link between start-up preparation and actual action.

Third, our study only used the questionnaire to collect data. Our analysis mainly revealed the indirect relationship between personality traits and start-up preparation. However, the mechanism behind the relationships was not well revealed and explained. Therefore, further research can adopt both qualitative and quantitative methods to underpin the mechanism. For example, interviews can be conducted to explain those non-significance relationships and those relationships that are not consistent with prior studies.

## Data availability statement

The raw data supporting the conclusions of this article will be made available by the authors, without undue reservation.

## Ethics statement

Written informed consent was obtained from the individual(s) for the publication of any potentially identifiable images or data included in this article.

## Author contributions

JZ: write the original manuscript, data collection and analysis, and discussion. RX: data collection. HS: review the manuscript. All authors contributed to the article and approved the submitted version.

## Funding

This paper was supported by the Strategic Public Policy Research Funding Scheme from the Policy Innovation and Co-ordination Office of the Government of the Hong Kong Special Administrative Region (S2020.A1.033.20S) and matching fund by the City University of Hong Kong (9678240).

## Conflict of interest

The authors declare that the research was conducted in the absence of any commercial or financial relationships that could be construed as a potential conflict of interest.

## Publisher’s note

All claims expressed in this article are solely those of the authors and do not necessarily represent those of their affiliated organizations, or those of the publisher, the editors and the reviewers. Any product that may be evaluated in this article, or claim that may be made by its manufacturer, is not guaranteed or endorsed by the publisher.
